# Exercise Questions on Chapter Fifth

**Published:** 1883-11

**Authors:** 


### EXERCISE QUESTIONS ON CHAPTER FIFTH.

---

What law of chemical combination was discovered at the dawn of the present century?

What more fundamental law was soon after announced by Dalton?

State the higher generalization of these laws?

What second law did Dalton announce?

What fourth and fifth laws were deduced from these?

Give in logical order the first law of chemical combination?

The second?

The third, fourth and fifth ?

What ancient theory did Dalton utilize in framing his doctrine of the atomic constitution of matter ?

To what in its definiteness may chemical action be compared ?

Give the illustration of multiple proportion ?

Illustrate each in succession ?

